# Rational Design of Key Enzymes to Efficiently Synthesize Phycocyanobilin in *Escherichia coli*

**DOI:** 10.3390/biom14030301

**Published:** 2024-03-03

**Authors:** Ziwei Wang, Jingwen Zhou, Jianghua Li, Guocheng Du, Jian Chen, Xinrui Zhao

**Affiliations:** 1 Science Center for Future Foods, Jiangnan University, 1800 Lihu Road, Wuxi 214122, China; 6210201041@stu.jiangnan.edu.cn (Z.W.); zhoujw1982@jiangnan.edu.cn (J.Z.); lijianghua@jiangnan.edu.cn (J.L.); gcdu@jiangnan.edu.cn (G.D.); 2 Key Laboratory of Industrial Biotechnology, Ministry of Education, School of Biotechnology, Jiangnan University, 1800 Lihu Road, Wuxi 214122, China; 3 Jiangsu Province Engineering Research Center of Food Synthetic Biotechnology, Jiangnan University, 1800 Lihu Road, Wuxi 214122, China; 4 Engineering Research Center of Ministry of Education on Food Synthetic Biotechnology, Jiangnan University, 1800 Lihu Road, Wuxi 214122, China; 5 Key Laboratory of Carbohydrate Chemistry and Biotechnology, Ministry of Education, Jiangnan University, 1800 Lihu Road, Wuxi 214122, China

**Keywords:** phycocyanobilin, biliverdin, *Escherichia coli*, rational design, high-throughput screening

## Abstract

Phycocyanobilin (PCB) is a natural blue tetrapyrrole chromophore that is found in phycocyanin and plays an essential role in photosynthesis. Due to PCB’s antioxidation, anti-inflammatory and anti-cancer properties, it has been utilized in the food, pharmaceutical and cosmetic industries. Currently, the extraction of PCB from *Spirulina* involves complex processes, which has led to increasing interest in the biosynthesis of PCB in *Escherichia coli*. However, the PCB titer remains low because of the poor activity of key enzymes and the insufficient precursor supply. Here, the synthesis of PCB was firstly improved by screening the optimal heme oxygenase (HO) from *Thermosynechococcus elongatus* BP-1(HO^T^) and PCB: ferredoxin oxidoreductase from *Synechocystis* sp. PCC6803 (PcyA^S^). In addition, based on a rational design and the infrared fluorescence method for high-throughput screening, the mutants of HO^T^(F29W/K166D) and PcyA^S^(D220G/H74M) with significantly higher activities were obtained. Furthermore, a DNA scaffold was applied in the assembly of HO^T^ and PcyA^S^ mutants to reduce the spatial barriers, and the heme supply was enhanced via the moderate overexpression of *hemB* and *hemH*, resulting in the highest PCB titer (184.20 mg/L) obtained in a 5 L fermenter. The strategies applied in this study lay the foundation for the industrial production of PCB and its heme derivatives.

## 1. Introduction

Phycocyanobilin (PCB) is a tetrapyrrole molecule found in blue algae and cryptophycean, and it plays a critical role in the transmission and absorption of light energy [1]. Recognized by the FDA as a “diamond food”, PCB has been utilized as a safe and nutritional pigment in the food and beverage industries [2]. In addition, PCB is extensively applied in cosmetics [3,4], healthcare products and pharmaceuticals due to its antioxidant, anti-inflammatory, anti-obesity and anti-diabetic properties [5,6]. In particular, it has been reported that PCB has potential therapeutic benefits for the treatment of nervous diseases [7,8]. Thus, there has been a growing demand for PCB in recent years. Currently, PCB is mainly obtained via the hydrolysis of *Spirulina* by methanol at 70–80 ℃ [9]. However, the methanolysis at high temperatures and the long growth cycle of *Spirulina* limit the large-scale production of PCB. Moreover, the presence of other similar pigments with PCB, including chlorophyll and lutein, makes the purification process difficult [10]. Furthermore, the chemical synthesis of PCB is restricted to regioselective and stereoselective catalysis [11]. Hence, more attention has been paid to the biosynthesis of PCB by microorganisms in recent years.

**Figure 1:**
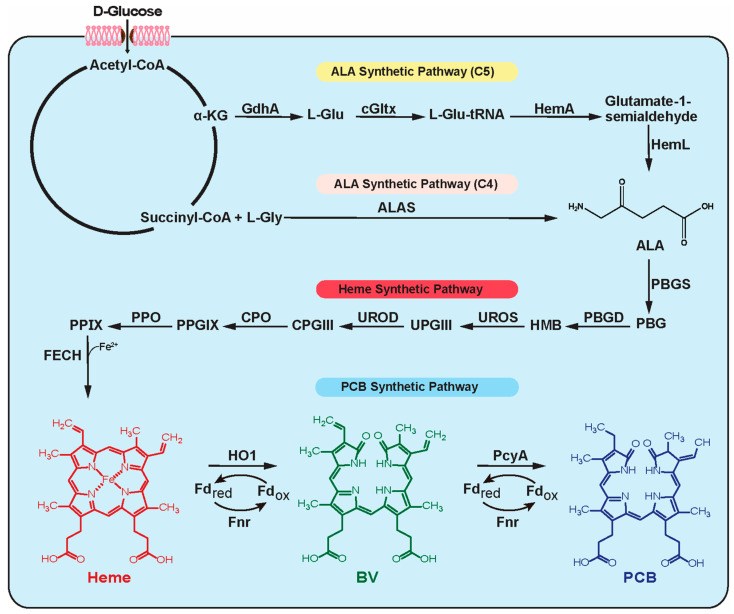
Schematic diagram of PCB biosynthesis. α-KG, α-ketoglutarate; GdhA, glutamate dehydrogenase; cGltx, glutamyl-tRNA synthetase; HemA, glutamyl-tRNA reductase; HemL, glutamate-1-semialdehyde 2,1-aminomutase; ALAS, 5-aminolevulinate synthase; 5-ALA, 5-aminolevulinic acid; PBG, porphobilinogen; PBGS, porphobilinogen synthase; HMB, 1-hydroxymethylbilane; PBGD, porphobilinogen deaminase; UPGIII, uroporphyrinogen III; UROS, uroporphyrinogen-III synthase; CPGIII, coproporphyrinogen III; UROD, uroporphyrinogen-III decarboxylase; PPGIX, protoporphyrinogen IX; CPO, coproporphyrinogen-III oxidase; PPIX, protoporphyrin IX; PPO, protoporphyrinogen oxidase; FECH, ferrochelatase; HO, heme oxygenase; BV, biliverdin; PcyA, phycocyanobilin: ferredoxin oxidoreductase; PCB, phycocyanobilin; Fd_red_, reduced ferredoxin; Fd_ox_, oxidized ferredoxin; Fnr, Fd-NADP^+^ reductase.

The biosynthesis of PCB begins with its important precursor ALA. In different species, ALA (5-aminolevulinic acid) is synthesized through the C4 or C5 pathway. ALA synthase (ALAS) catalyzes the conversion of L-glycine and succinyl-coenzyme A (succinyl-CoA) into ALA via the C4 pathway, which is found in animals and fungi. On the other hand, plants and most bacteria utilize the C5 pathway to convert L-glutamate into ALA through a series of reactions catalyzed by glutamyl-tRNA synthetase (cGltx), glutamyl-tRNA reductase (HemA) and glutamate-1-semialdehyde 2,1-aminomutase (HemL) [12]. After ALA is synthesized, it is transformed into heme with a series of enzymes, including porphobilinogen synthase (PBGS), porphobilinogen deaminase (PBGD), uroporphyrinogen-III synthase (UROS), uroporphyrinogen-III decarboxylase (UROD), coproporphyrinogen-III oxidase (CPO), protoporphyrinogen oxidase (PPO) and ferrochelatase (FECH) [13]. Then, heme oxygenase (HO) catalyzes the oxidization of heme to synthesize biliverdin (BV) by using oxygen and reduced ferredoxin [14]. Finally, phycocyanobilin: ferredoxin oxidoreductase (PcyA) reduces two vinyl groups of BV to form PCB by utilizing ferredoxin [15] (Figure 1).

Among various microbial hosts, *Escherichia coli*, as a microbial cell factory, possesses numerous advantages for the production of natural products [16]. As for the biosynthesis of PCB, Gambetta et al. successfully transformed the *ho1* and *pcyA* genes from *Synechocystis sp.* PCC6803 in *E. coli*, leading in the initial production of PCB in vitro [17]. In addition, Ge et al. further improved the biosynthesis of PCB via modular metabolic engineering, resulting in a yield of 6.64 mg/L at the shaking-flask level [9]. In the following, an optogenetic-driven transcriptional regulation system (PhyB-PIF LID) was developed to co-localize the expression of HO1 and PcyA [18]. Then, an optimized strategy was developed for the fermentation of PCB in *E. coli* (TB medium, 4.00 mM lactose induction, an induction temperature of 24.69 °C, an induction time of 4.60 h and an induction duration of 13.57 h), producing 13.00 mg/L of PCB at the shaking-flask level [19]. Moreover, Zhao et al. improved the reducing power by constructing a cofactor circulation system, overexpressing the *nadK* gene (encoding NAD^+^ kinase) and assembling HO1 and PcyA by a DNA scaffold, producing 28.32 mg/L of PCB in a 5 L fermenter [20]. In the latest research, Ho1 and PcyA were assembled by an RIAD-RIDD scaffold to increase their catalytic efficiency, and the endogenous production of heme (precursor) was improved via the integrated expression of the key genes *hemB*, *hemC*, *hemD*, *hemE*, *hemF*, *hemG* and *hemH* in the genome, which achieved 147.00 mg/L of PCB [21]. Although the PCB titer gradually increased, there are still many problems that need to be solved in the biosynthesis of PCB in *E. coli*. The activities of the key enzymes responsible for PCB synthesis remain low, which greatly weakens the biosynthetic efficiency.

In this study, the optimal rate-limiting enzymes (HO and PcyA) for PCB synthesis were first selected from different sources. In the following, rational modifications of HO^T^ and PcyA^S^ were performed, and the most effective mutants of these two enzymes were identified based on the high-throughput screening assay. Then, DNA scaffolds were used to assemble HO^T^(F29W/K166D) and PcyA^S^(D220G/H74M), and the key enzymes responsible for heme synthesis were overexpressed to provide enough precursor for PCB synthesis. Finally, the highest PCB titer was achieved at the 5 L bioreactor level.

## 2. Materials and Methods

### 2.1. Strains and Media

The strains applied and constructed in this study are listed in Table S1*. E. coli* DH5α and BL21(DE3) were used as hosts for DNA cloning and PCB synthesis, respectively. LB, TB, MR and GMD-Gly media were tested to synthesize PCB. After the optimal conditions were confirmed, the GMD-Gly medium, with 5 g/L (NH_4_)_2_SO_4_, 10 g/L maltodextrin, 0.10 g/L vitamin B1, 5 g/L ascorbic acid and 1 mL/L trace elements, was used for fed-batch fermentation [21]. All reagents not described above were purchased from Sangon Biotech Co., Ltd. (Shanghai, China).

### 2.2. Plasmids and DNA Manipulations

The genes and primers used in this study are listed in Tables S2 and S3. The heterologous genes, including *ho^T^* from *Thermosynechococcus elongatus* BP-1, *ho^S^* from *Synechocystis* sp. PCC6803, *ho^N^* from *Nostoc* sp. PCC 7120, *ho^NM^* from *Neisseria meningitidis*, *ho^GM^* from *Glycine max*, *ho^H^* from *Homo sapiens*, *ho^B^* from *Bos taurus*, *ho^O^* from *Oryctolagus cuniculus*, *ho^R^* from *Rattus norvegicus*, *ho^GG^* from *Gallus gallus*, *pcyA^S^* from *Synechocystis sp.* PCC6803, *pcyA^T^* from *Thermosynechococcus elongatus* BP-1, *pcyA^N^* from *Nostoc sp.* PCC 7120, *pcyA^SU^* from *Synechococcus* sp., *pcyA^P^* from *Prochlorococcus sp*., *ADB1* and *ADB2* encoding zinc finger proteins, *iRFP* and *Alr1966g2C56A* encoding infrared fluorescence proteins were codon-optimized and synthesized by GenScript Biotech Co., Ltd. (Nanjing, China). The other genes, including *hemB*, *hemH* and constitutive promoters (PnudC), were amplified from the genome of *E. coli* BL21(DE3). Oligonucleotide primers were synthesized by Sangon Biotech Co., Ltd. (Shanghai, China).

To promote the soluble expression of HO^N^, HO^GG^, PcyA^SU^ and PcyA^P^, *MBP* was, respectively, amplified using the primers F1/R1, F1/R1, F2/R2 and F3/R3; and sequentially fused with *ho^N^*, *ho^GG^*, *pcyA^SU^* and *pcyA^P^* and inserted into pRSFDuet-1 plasmids (the plasmids were amplified using the primers F4/R4, F5/R4, F6/R5 and F7/R6, respectively); and transformed to the *E. coli* BL21(DE3) host to obtain the engineered strains S3, S10, S12 and S13.

To construct a large number of HO and PcyA mutants, base substitutions were introduced to the plasmids using primers (with the desired mutation) according to a PCR protocol that amplifies the entire plasmid. The two primers had a 15 bp overlap at their 5′ ends so that the PCR products could anneal to each other after amplification.

To assemble HO^T^(F29W/K166D) and PcyA^S^(D220G/H74M) by DNA scaffolds, *ADB1* and *ADB2* were fused to *ho^T^*(*F29W/K166D*) *and pcyA^S^*(*D220G/H74M*) via two rounds of PCR using the primers F8/R7 and F9/R8, respectively. DNA scaffold1 (binding the ADB1 domain) and DNA scaffold2 (binding the ADB2 domain) were added to the pCDFDuet-1 plasmids at a 2:1 ratio using F10/R9.

To increase the precursor supply (heme), the linearized pCDFDuet-T7*lac*-scaffold1-scaffold1-scaffold2 plasmid and weak constitutive promoter (PnudC) were amplified using the primers F11/R10 and F12/R11 to replace the second T7*lac* promoter in the plasmid. Then, the *PBGS* and *FECH* genes were amplified using the primers F13/R12 and F14/R13, and they were inserted into the pCDFDuet-T7*lac*-scaffold1-scaffold1-scaffold2 plasmid between the *Nde* I/*Bgl* II and *Bgl* II/*Xho* I sites, respectively.

### 2.3. Culture Conditions

For the synthesis of BV/PCB at the shaking-flask level, the seed was incubated at 37 °C and 220 rpm for 12 h. Then, the culture was transferred to the fermentation medium at an inoculum level of 2%. When the value of OD_600_ reached 0.60–0.80, 0.50 mM isopropyl-β-D-thio-galactopyranoside (IPTG) was added to induce the synthesis of PCB at 30 °C.

Fed-batch fermentations were performed using a 5 L bioreactor (T&J Bioengineering, Shanghai, China). An amount of 0.15 L of secondary seed culture was inoculated into a fermenter containing 2.35 L of the fermentation medium. During the early stages, the temperature was maintained at 37 °C. IPTG was added when a rebound of dissolved oxygen (DO) appeared, at which time the temperature was cooled to 30 °C. In the process of fermentation, the pH value was maintained at 7.00 via the automatic addition of 50% NH_4_OH. The air flow rate was set to 1 vvm, and agitation was maintained from 400 rpm to 600 rpm. The DO-stat strategy was applied to maintain the dissolved oxygen level at 40%. The feed solution contained 600 g/L glycerol, 0.20 g/L FeSO_4_·7H_2_O, 6.25 g/L (NH_3_)_4_SO_4_, 15 g/L MgSO_4_·7H_2_O, 5 g/L ascorbic acid and 0.10 g/L VB1, and the gradient of the feeding speed was as follows: 12–16 h, 20 mL/h; 16–20 h, 22 mL/h; 20–24 h, 25 mL/h; 24–28 h, 27 mL/h; 28–32 h, 30 mL/h; 32–36 h, 27 mL/h; 36–40 h, 25 mL/h; 40–44 h, 22 mL/h; 44–48 h, 20 mL/h; 48–52 h, 18 mL/h; 52–56 h, 15 mL/h; and 56–60 h, 12 mL/h.

### 2.4. Preparation of Detection Reagents (BDR/PDR) and Standard Curves of BV and PCB for High-Throughput Screening Assays

The S16 and S17 strains were incubated in 2 mL of LB medium at 37 °C and 220 rpm for 10–12 h; then, the seed solution was transferred to 25 mL of LB medium at an inoculum volume of 2% and incubated at 30 °C and 220 rpm in the dark for 10–12 h. Next, the fermentation broth was diluted using ultrapure water to OD_600_ = 2.50 and centrifuged at 8000 g. The precipitate was suspended in PBS buffer (pH = 7.20) and put on ice for 10 min (2 s on/4 s off pulse cycles) of ultrasonic disruption. Finally, the bacterial lysate was centrifuged at 11,000 g and 4 °C for 20 min. The supernatant (BDR/PDR) was aliquoted and stored at −40 °C for the high-throughput screening of the BV and PCB samples.

To prepare a standard curve of BV, the BV concentration gradients were chosen as follows: 1, 5, 10, 20, 25, 40 and 50 mg/L. Then, 10 μL of BV standard solution was mixed with 90 μL of BV detection reagent (BDR) in a black flat-bottomed 96-well plate and incubated for 15 min at indoor temperature. The fluorescence intensity was measured by a microplate reader at 665/725 nm, with the max simulation gain [22]. Similarly, to prepare a standard curve of PCB, 80 μL of PCB standard solution with various concentration gradients (1, 5, 10, 25 and 50 mg/L) and 20 μL of PCB detection reagent (PDR) were added to a black 96-well plate. Following a 15 min incubation period at ambient temperature, the fluorescence was quantified at 546/650 nm [23]. The samples were measured three times, and the fluorescence value was repeatedly recorded for each well.

### 2.5. Analytical Procedures

The BV standard (>98%) and PCB standard (>95%) were purchased from Beiyu Biotechnology Co., Ltd. (Nanjing, China) and Frontier Specialty Chemicals, respectively. For the preparation of the BV samples and the PCB samples, a fermentation broth was mixed with methanol and shaken for 5 min. Then, the mixture was centrifugated at 13,000 g for 15 min. The supernatant (filtrated through a 0.22 μm filter membrane) was taken for an HPLC analysis (Shimadzu Corporation, Kyoto, Japan) using a Robussil C18 Plus column (5 μm, 4.60 mm × 250 mm). The column temperature was maintained at 30 °C. For the analysis of BV, a flow rate of 1 mL/min was used in an isocratic elution method: methanol- ultrapure water (pH = 2.60 via the adjustment of acetic acid) = 70:30. The absorbance was monitored at 376 nm and the retention time was 12.69 min. For the analysis of PCB, a flow rate of 0.80 mL/min was used in a gradient elution method: 40% buffer A (0.10% trifluoroacetic acid in ultrapure water) and 60% buffer B (0.10% trifluoroacetic acid in acetonitrile) at 0 min, and 45% buffer A and 55% buffer B at 15 min. The absorbance was monitored at 380 nm and the retention time was 11.51 min.

### 2.6. Homology Modeling and Ligand Docking

A homology model of HO^T^ was constructed using the template of HO^S^ (PDB: 1WE1, 71.40% sequence identity, 86.60% sequence similarity, 2.50 Å) in Discovery Studio 2019 (DS 2019) [24,25]. Twenty predicted atom models were scored, and the loop-region, main chain and side chain were refined; the HO^T^ model with a −25997 of DOPE SCORE and 98.50% residues in the allowed region was selected as the best model. As a molecular docking method based on CHARMm’s position, CDOCKER can produce highly accurate docking results for various types of covalent and non-covalent interactions, and it has been successfully applied in the docking of small-molecule ligands to hemoproteins, such as cytochrome P450 [26,27,28]. The analysis of molecular docking in this study was performed using the CDOCKER tool in DS 2019.

## 3. Results and Discussion

### 3.1. Screening of the Optimal HO Source for the Synthesis of BV

Biliverdin (BV) is an important intermediate in the synthesis of PCB. To improve the production of PCB, it is necessary to increase the accumulation of BV. The key heme oxygenase (HO) catalyzes the degradation of heme into BV [29]. Although the heterologous biosynthesis of BV has been achieved by expressing HO in *E. coli*, there has been no comparative study of HO activities among different species, resulting in a relatively low BV titer [30]. Therefore, based on the BRENDA enzyme database, ten different sources of HO were investigated with the aim of obtaining higher HO activity for the synthesis of BV. At first, ten engineered strains were constructed to express these enzymes from different sources.

**Figure 2:**
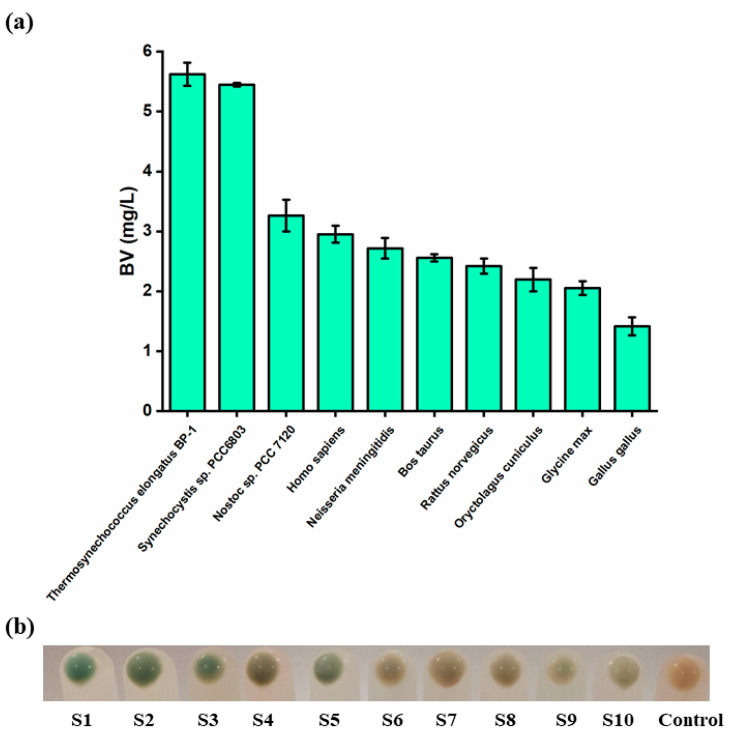
Screening of the optimal HO source for the synthesis of BV. (**a**) Production of HO from different sources. (**b**) The cell pellets of S1–S10 strains. The last one is the control group. Values and error bars represent means and standard deviations of biological triplicates.

As shown in Figure 2a, compared to the other strains, the S1 strain harboring *ho^T^* from *Thermosynechococcus elongatus* BP-1 produced a higher BV titer (5.62 mg/L). However, there is a significant difference between the S1 strain and the S9 and S10 strains in the biosynthesis of BV, indicating the lower activity of HO from plants and mammals in *E. coli* (Figure 2b). The expression of HO from eukaryotes is probably unsuitable for BV synthesis in *E. coli*. Thus, the S1 strain harboring HO^T^ was chosen as the optimal enzyme for further studies.

### 3.2. Development of a High-Throughput Assay for the Detection of BV

After determining the most efficient source of HO, it is urgent to develop a high-throughput assay to detect BV for the screening of HO with higher activity in the following. In the past, high-performance liquid chromatography (HPLC) has been frequently applied to detect BV, but it has many limitations, including high costs and time-consuming operations. It has been reported that a bacterio-phytochrome, RpBphP210, derived from the photosynthetic bacterium *Rhodopseudomonas palustris*, was modified to form a near-infrared fluorescent protein called iRFP (35.80 kDa). The PAS and GAF domains could covalently bind with BV and emit infrared light. Compared with the previously used photochrome fluorescent probe, iRFP exhibits a more effective brightness and intracellular and extracellular photostability. Thus, iRFP has been used as a typical GFP-like protein [31].

**Figure 3:**
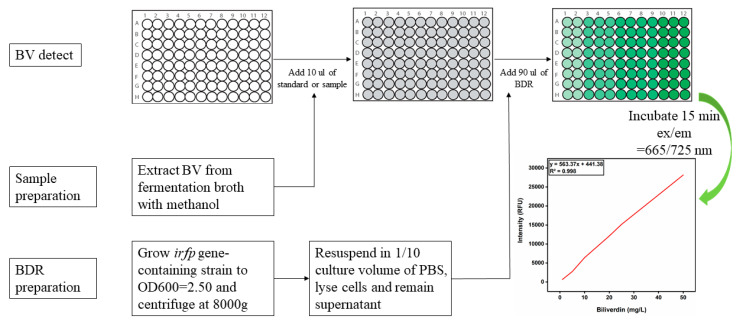
High-throughput assay using iRFP to detect BV. The standard is BV standard solution with different concentration gradients (1, 5, 10, 20, 25, 40 and 50 mg/L). The sample is prepared by mixing fermentation broth with isopycnic methanol. For the preparation of BDR, grow *irfp* gene-containing strain to OD_600_ = 2.50 and centrifuge at 8000 g, resuspend in 1/10 culture volume of PBS, lyse cells and remain supernatant. For the same batch of samples, the same BDR solution should be used, and the prepared BDR should be stored at −40 ℃.

Here, a high-throughput screening assay was designed using iRFP, and it was applied to determine the BV titer and screen the most effective HO. Firstly, the prepared standard curve of BV demonstrated that the fluorescence value exhibited a linear relationship within the detection range of 1–50 mg/L, with the R^2^ exceeding 0.99 (Figure 3). Next, the accuracy of the method was evaluated by testing the fluorescence value of 100 uL of the BV standard solution without the addition of BDR. The results show that, with the increase in the BV concentration, the fluorescence values did not linearly increase at 665/725 nm, indicating that the existence of BDR is essential for detection. Furthermore, to avoid the interference of heme in the detection of BV, different concentrations of heme were added to the mixed samples of BDR and the BV standard solution, and it was found that the heme concentration had no significant effect on the fluorescence value and did not affect the detection of BV.

Finally, the fermentation broths of the S1–S10 strains were mixed with methanol and incubated with BDR, the fluorescence values were measured and the BV titers were calculated. The results of the BV synthesis determined through the novel method were consistent with the results of the HPLC method, further proving the usability of a high-throughput assay with iRFP.

### 3.3. Improving the Catalytic Activity of HO by Rational Design

Considering that the level of the obtained BV was still low, the stability of HO and the affinity between HO and the substrate (heme) were enhanced by rational design. The modifications were based on the homology structure model of HO^T^, which was constructed using the highly homologous template of HO^S^ (PDB: 1WE1, 71.40% sequence identity, 86.60% sequence similarity and 2.50 Å). An analysis of the molecular docking between the model of HO^T^ and heme was performed, and the amino acids within 5 Å around the active center were subjected to alanine scanning and virtual saturation mutations. Mutants with an energy of less than −0.50 kcal/mol were selected for construction.

**Figure 4:**
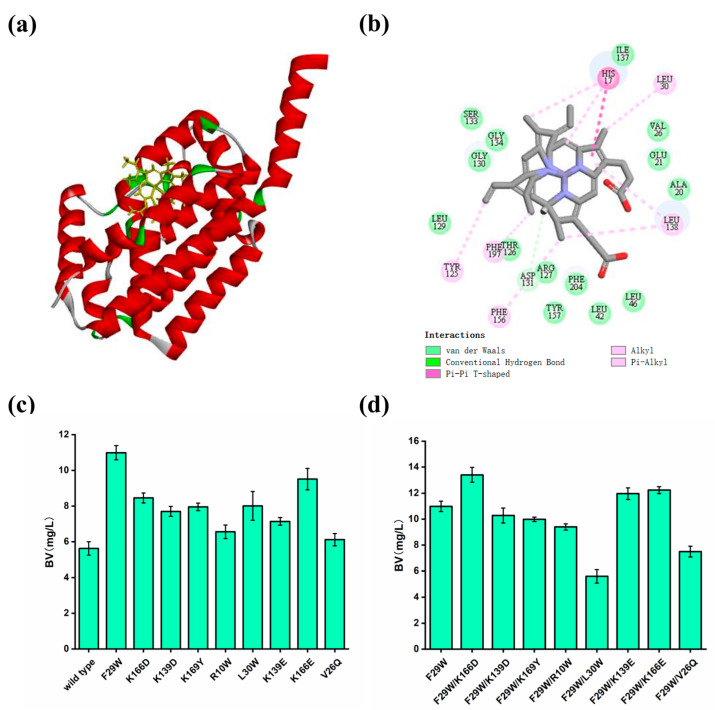
Improving the catalytic activity of HO by rational design. (**a**) Molecular docking between HO^T^ model and heme. (**b**) The interactions between heme and HO^T^ model. (**c**) Titers of BV produced by engineering strains containing HO^T^ single mutants. (**d**) Titers of BV produced by engineering strains containing HO^T^ double mutants. Values and error bars represent means and standard deviations of biological triplicates.

Compared to the wild-type HO^T^, nine single mutants exhibited increased catalytic activity (F29W, K166D, K139D, K169Y, R10W, L30W, K139E, K166E and V26Q). Among them, HO^T^(F29W) had the highest BV titer (10.99 mg/L), which increased by 95.55%. Then, eight double mutants (F29W/K166D, F29W/K139D, F29W/K169Y, F29W/R10W, F29W/L30W, F29W/K139E, F29W/K166E and F29W/V26Q) were constructed, and the yield of HO^T^ (F29W/K166D) was further improved by 22.02%, producing 13.41 mg/L of BV (Figure 4).

To analyze the reasons for the increased titer, a homology model of the double-mutant HO^T^ (F29W/K166D) (distance = 2.11 Å, angle DHA = 141.48 and angle HAY = 153.33) was constructed and examined. Subsequently, with heme as the ligand, CDOCKER was used to perform an analysis of the molecular docking. According to the docking results, although the amino acids Trp29 and Asp166 did not directly interact with the substrate heme, the hydrogen bonds around the heme increased in the HO^T^ (F29W/K166D) model compared to those in the wild-type HO^T^ model. In addition, the increased hydrophobic interaction of Trp29 and Asp166 with the surrounding amino acids may lead to a more flexible access to heme (Supplementary Figure S1).

### 3.4. Screening of the Ideal PcyA Source for the Synthesis of PCB

After obtaining the best engineered HO, improving the catalytic activity of phycocyanobilin: ferredoxin oxidoreductase (PcyA) becomes an essential step to increase PCB synthesis [32]. PcyA is a member of the FDBR family found in algae. Contrary to other FDBRs, PcyA does not rely on any metal ion or organic cofactor. Instead, it utilizes four electrons from ferredoxin to facilitate the reduction of the d-ring and a-ring vinyl groups of BV, further leading to the synthesis of PCB. Similar with HO, the activity of PcyA from various algae has not yet been determined. Therefore, according to the BRENDA enzyme database, five PcyAs from different algae were selected, and five engineered strains were constructed to obtain higher activity for PCB synthesis.

**Figure 5:**
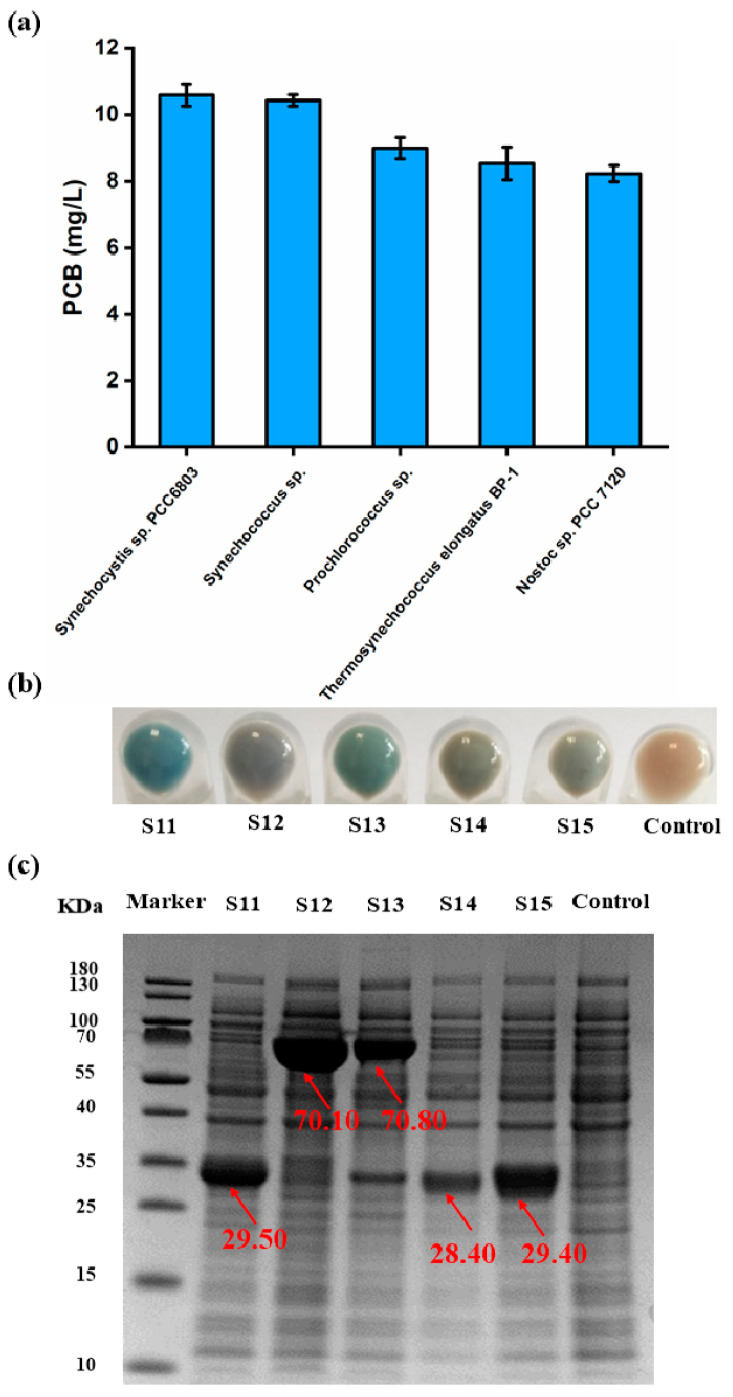
Screening of the optimal PcyA source for the synthesis of PCB. (**a**) Production of PcyA from different sources. (**b**) The cell pellets of S11–S15 strains. The last one is the control group. (**c**) SDS-PAGE of S11–S15 strains (the original images can be found in Figure S2). The molecular weight of PcyA^S^, MBP-PcyA^SU^, MBP-PcyA^P^, PcyA^T^ and PcyA^N^ containing 10 × His tags was 29.50, 70.10, 70.80, 28.40 and 29.40 KDa, respectively. Values and error bars represent means and standard deviations of biological triplicates.

The results revealed that the S11 strain containing *pcyA^S^* from *Synechocystis* sp. PCC6803 exhibited a greater PCB titer (10.60 mg/L), which was visually confirmed by its brilliant blue color (Figure 5a,b). However, compared to S11 and S12, S13 exhibited a higher degree of green coloration (Figure 5b), which indicates that the BV substrate was in surplus and that the yield of PCB was limited. Furthermore, it was found that the fusion of MBP with PcyA^SU^ and PcyA^P^ increased the solubility of proteins, but their catalytic activities were still low in *E. coli* (Figure 5c) (Supplementary Figure S2). Thus, PcyA^S^ was determined as the ideal enzyme for the subsequent experiments.

### 3.5. Establishment of a High-Throughput Assay for the Detection of PCB

Since the optimal source of PcyA was identified and HPLC is not suitable for screening a large number of PcyA mutants, a high-throughput assay was established for the rapid detection of PCB. In a previous study, researchers obtained a photosensitive protein mutant, Alr1966g2C56A, belonging to the cyanobacterial family of pigments (CBCR) from the freshwater alga Nostoc sp. PCC 7120. The sulfur atom in the conserved cysteine residue at position 84 of Alr1966g2C56A creates a thioether bond with the carbon atom of PCB in the GAF domain, allowing for the stable attachment of PCB to Alr1966g2C56A [33].

**Figure 6:**
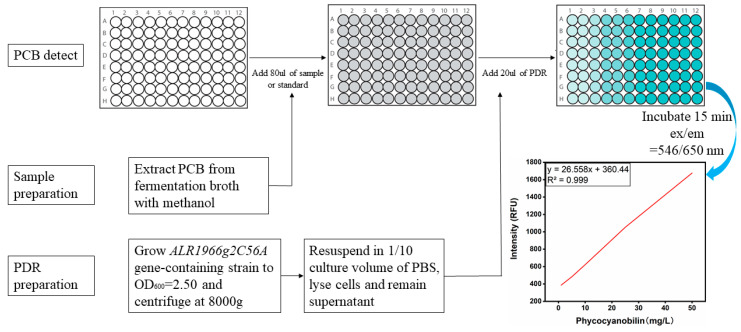
High-throughput assay using Alr1966g2C56A to detect PCB. The standard is PCB standard solution with different concentration gradients (1, 5, 10, 25 and 50 mg/L). The sample is prepared by mixing fermentation broth with isopycnic methanol. For the preparation of PDR, grow *Alr1966g2C56A* gene-containing strain to OD_600_ = 2.50 and centrifuge at 8000 g, resuspend in 1/10 culture volume of PBS, lyse cells and remain supernatant. For the same batch of samples, the same PDR solution should be used, and the prepared PDR should be stored at −40 ℃.

Based on the specific combination of PCB and Alr1966g2C56A, an efficient and convenient high-throughput assay was established to measure the PCB titer. As shown in the standard curve of PCB, within the detection limit of 1–50 mg/L, an upward trend in the fluorescence value was revealed, with a value of R^2^ higher than 0.99 (Figure 6). To validate the accuracy of the method, the fluorescence values of 100 uL of the PCB standard solution (1, 5, 10, 25 and 50 mg/L) were monitored without the addition of PDR. The observed fluorescence values at 546/650 nm were low and did not linearly increase with the increase in the PCB concentration, indicating that PDR is indispensable for detection. Next, 100 uL of PDR was mixed with various concentrations of heme standard solutions (0.1, 1, 10, 100, 500 and 1000 mg/L) and BV standard solutions (1, 5, 10, 25, 50 and 100 mg/L). The results showed that the fluorescence values formed by PDR and PCB were unaffected by heme and BV (Figure 6).

Subsequently, the fermentation broths of the S11–S15 strains were mixed with methanol and tested after incubation with PDR. The PCB titers produced by S11–S15 and measured by the high-throughput assay were in accordance with those measured by HPLC. Not only can the novel high-throughput assay dramatically decrease the detection time of PCB and the complexity of operation, but it can also be employed to efficiently screen PcyA in order to enhance the synthetic efficiency of PCB. Additionally, it provides valuable insights into the development of detection methods for other chromophores.

### 3.6. Enhancing the Catalytic Activity of PcyA by Rational Design

Since the level of synthesis of PCB was still low, the catalytic activity of PcyA also had to be improved. Based on the selection of PcyA from different sources, PcyA^S^ (PDB: 2D1E, 1.51 Å) was used for molecular docking with BV [34]. Alanine scanning and virtual saturation modifications were performed on the amino acids located within a 7 Å radius from the active center. The mutants with an energy below −0.70 kcal/mol were picked for construction.

**Figure 7:**
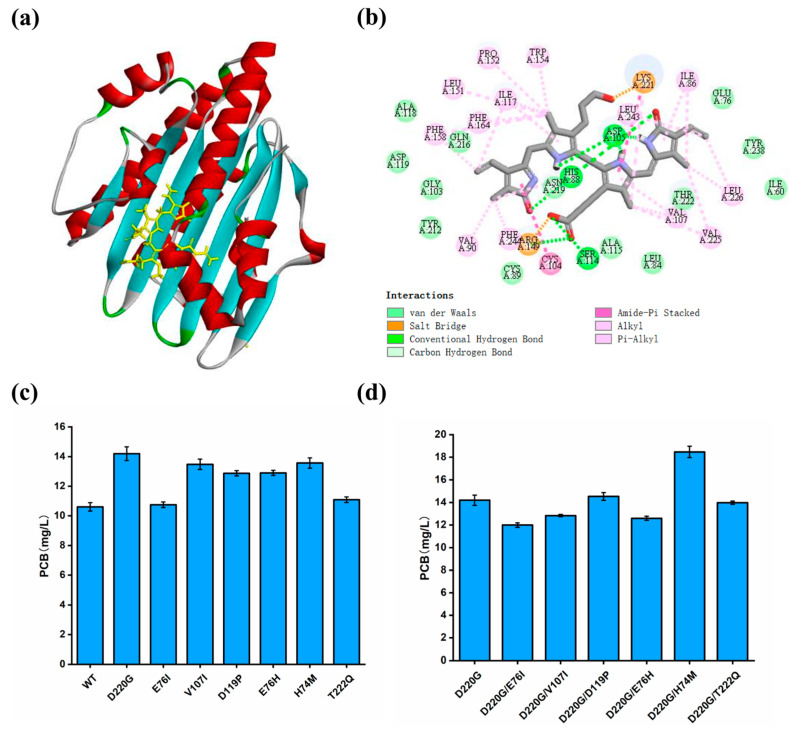
Improving the catalytic activity of PcyA by rational design. (**a**) Molecular docking between PcyA^S^ and BV. (**b**) The interactions between BV and PcyA^S^. (**c**) Titers of PCB produced by engineering strains containing PcyA^S^ single mutants. (**d**) Titers of PCB produced by engineering strains containing PcyA^S^ double mutants. Values and error bars represent means and standard deviations of biological triplicates.

Among the single mutants, PcyA^S^ (D220G) achieved the highest PCB titer of 14.20 mg/L, which was increased by 33.96% compared to the wild-type PcyA^S^. Based on PcyA^S^ (D220G), six double mutants PcyA^S^ (D220G/E76I, D220G/V107I, D220G/D119P, D220G/E76H, D220G/H74M and D220G/T222Q) were constructed, but only the yield of PcyA^S^ (D220G/H74M) was further increased by 30.07%, producing 18.47 mg/L of PCB (Figure 7).

To analyze the reasons for the increased titer, a homology model of the double-mutant PcyA^S^ (D220G/H74M) (distance = 1.86 Å, angle DHA = 157.26 and angle HAY = 105.71) was constructed and examined. By applying BV as the ligand, CDOCKER was used to carry out a molecular docking analysis between the enzyme and substrate. The results demonstrated that, although the BV substrate did not directly interact with the amino acids Met74 and Gly220, the electrostatic interactions around BV increased in the PcyA^S^ (D220G/H74M) model compared to those in the wild-type PcyA^S^ model. In addition, the increased hydrophobic interaction of Met74 and Gly220 with the surrounding amino acids may lead to a more flexible access to BV (Supplementary Figure S3).

### 3.7. Assembling of Key Enzymes and Enhancement in Heme Supply to Improve PCB Synthesis

**Figure 8:**
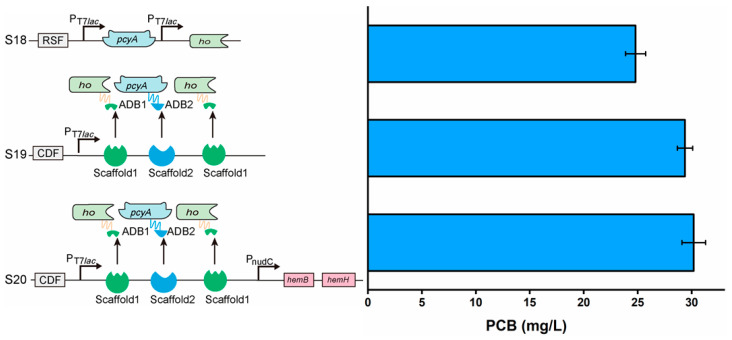
Assembly of enzymes and supplement of precursor to improve PCB synthesis. ADB1 and ADB2 are two zinc finger proteins that can specially bind to DNA scaffold1 and scaffold2. Values and error bars represent means and standard deviations of biological triplicates.

Since HO^T^(F29W/K166D) and PcyA^S^(D220G/H74M) were chosen as the best enzymes for PCB synthesis, these two enzymes were used together in the S18 strain (harboring pRSFduet-T7*lac-ho^T^*(*F29W/K166D*)-T7*lac-pcyA^S^*(*D220G/H74M*)). Compared with the S11 strain, the PCB titer (24.80 mg/L) was significantly improved by the S18 strain (Figure 8). To further improve PCB synthesis, attention should be paid to the distance between HO and PcyA. In recent years, it has been reported that an artificial scaffold can shorten the spatial distance between key enzymes [35]. Among common artificial scaffolds, DNA scaffolds have become a hotspot and have improved the production of various metabolites, including resveratrol, 1,2-propanediol, mevalonate and N-acetylglucosamine [36,37]. Thus, a DNA scaffold was used to assemble HO^T^(F29W/K166D) and PcyA^S^(D220G/H74M).

Furthermore, by fine-tuning the DNA scaffold to achieve a 2:1 binding ratio of HO^T^(F29W/K166D) and PcyA^S^(D220G/H74M), it is possible to enhance the local concentration of biliverdin intermediates during multi-enzyme reactions. Therefore, a new engineered strain (the S19 strain, harboring pRSFduet-T7*lac-ADB1-ho^T^*(*F29W/K166D*)-T7*lac-ADB2-pcyA^S^*(*D220G/H74M*) and the pCDFDuet-T7*lac-scaffold-scaffold2-scaffold1* plasmid) was constructed using DNA scaffolds, resulting in an increased PCB titer to 29.56 mg/L (Figure 8).

Following the enhancement in the catalytic efficiency of HO and PcyA, it is essential to ensure an adequate supply of heme (precursor) due to its low natural content in *E. coli* [38]. However, the exogenous addition of heme is not efficient and has toxic effects on the host [39]. Therefore, two key enzymes in the heme synthetic pathway (PBGS and FECH) were overexpressed by the weak constitutive promoter *nudC* to moderately enhance the heme supply. The S20 strain (harboring pRSFduet-T7*lac-ADB1-ho^T^*(*F29W/K166D*)-T7*lac-ADB2-pcyA^S^*(*D220G/H74M*) and the pCDFDuet-T7*lac-scaffold1-scaffold2-scaffold1*-PnudC-*PBGS*-*FECH* plasmid) was constructed, and the PCB titer was further improved to 30.18 mg/L (Figure 8).

### 3.8. Scale-Up of Fed-Batch Fermentation in 5 L Fermenter

**Figure 9:**
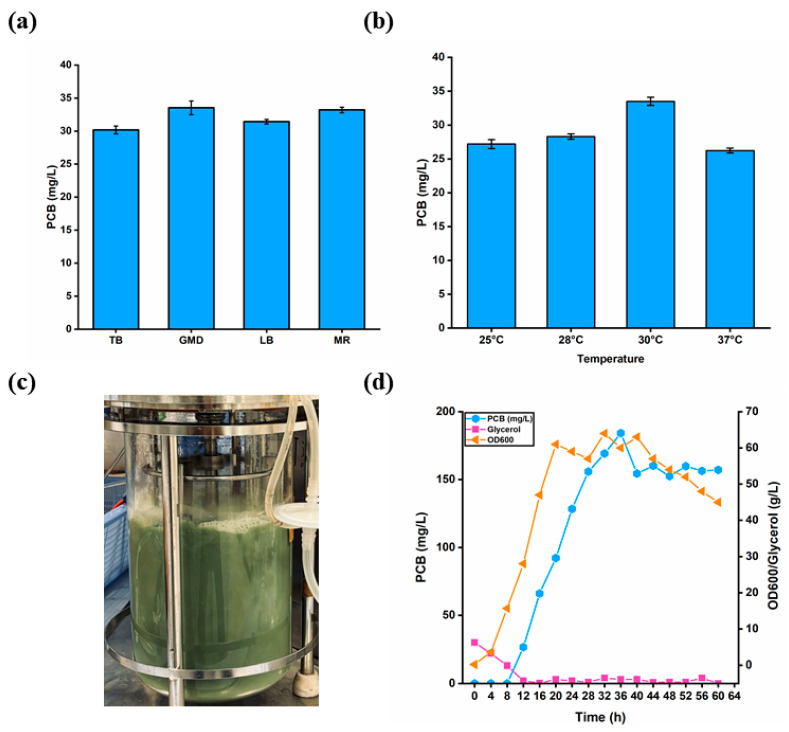
Optimization of fermentation conditions at shaking-flask level and 5 L fermenter level. (**a**) The selection of optimal fermentation medium at shaking-flask level. (**b**) The selection of suitable induction temperature at shaking-flask level. (**c**) The S20 strain fermented in GMD-Gly fermentation medium at 36 h in 5 L bioreactor. (**d**) Fed-batch fermentation by feeding with 600 g/L glycerol, 0.20 g/L FeSO_4_·7H_2_O, 6.25 g/L (NH_3_)_4_SO_4_, 15 g/L MgSO_4_·7H_2_O, 5 g/L ascorbic acid and 0.10 g/L VB1. Induction when DO picks up. The blue circles designate the concentrations of PCB, the orange triangles represent the values of OD_600_ and the pink squares indicate the remaining amount of glycerol. Values and error bars represent means and standard deviations of biological triplicates.

After obtaining the optimal engineered strain (the S20 strain), the proper fermentation conditions for PCB synthesis at the shaking-flask level were confirmed in the following, including the different media (TB, GMD, LB and MR) and induction temperatures (25 °C, 28 °C, 30 °C and 37 °C). As shown in Figure 9a,b, the GMD medium and induction at 30 °C were the best conditions for PCB synthesis, and the PCB titer reached 33.50 mg/L at 24 h.

Subsequently, based on the optimized fermentation conditions, fed-batch fermentation was performed for the S20 strain in a 5 L fermenter. When the optical density (OD) reached 50, the temperature was lowered to 30 °C, and IPTG was added to induce the synthesis of PCB. To further increase PCB synthesis, the DO-stat method was applied to maintain the DO value at 40. Finally, the maximum PCB titer reached 184.20 mg/L at 36 h (Figure 9c,d).

## 4. Conclusions

In this study, the catalytic activities of HO and PcyA, obtained from diverse sources, were firstly compared in *E. coli*. Then, mutants of HO^T^ (F29W/K166D) and PcyA^S^ (D220G/H74M) were obtained by rational design through high-throughput assays to significantly improve PCB synthesis. In addition, DNA scaffolds were used to remove the spatial barrier between HO and PcyA. Furthermore, two key enzymes involved in the heme synthetic pathway (PBGS and FECH) were overexpressed to enhance the intracellular heme supply. Finally, a PCB titer of 184.20 mg/L was achieved in a 5 L fermenter, which is the highest level reported to date.

## Data Availability

The data presented in this study are available on request from the corresponding author.

## Supplementary Materials

The following are available online at https://www.mdpi.com/article/10.3390/biom14030301/s1, Table S1: The strains applied and constructed in this study; Table S2: Genes used in the present study; Table S3: The primers used in this study; Figure S1: The interactions between heme and HO^T^ model (a) and HO^T^ (F29W/K166D) model (b); Figure S2: SDS-PAGE of S11–S15 strains; Figure S3: the interactions between BV and PcyA^S^ model (a) and PcyA^S^ (D220G/H74M) model (b).
