# Supplementary material for: Rational Design of Key Enzymes to Efficiently Synthesize Phycocyanobilin in Escherichia coli
Source: Biomolecules. 2024 Mar 3;14(3):301. doi: 10.3390/biom14030301 (PMC10968380; doi:10.3390/biom14030301)
Supplement: Supplementary file 1 [file biomolecules-14-00301-s001.zip › biomolecules-2884220-supplementary.pdf]

Supporting Information

Rational design of key enzymes to efficiently synthesize phycocyanobilin in *Escherichia coli*

Ziwei Wang [1,2,3,4], Jingwen Zhou [1,2,3,4], Jianghua Li [1,2,3,4], Guocheng Du [1,2,3,4,5], Jian Chen [1,2,3,4*], Xinrui Zhao [1,2,3,4*]

[1] Science Center for Future Foods, Jiangnan University, 1800 Lihu Road, Wuxi, Wuxi 214122, China

[2] Key Laboratory of Industrial Biotechnology, Ministry of Education, School of Biotechnology, Jiangnan University, 1800 Lihu Road, Wuxi, Wuxi 214122, China

[3] Jiangsu Province Engineering Research Center of Food Synthetic Biotechnology, Jiangnan University, 1800 Lihu Road, Wuxi 214122, China

[4] Engineering Research Center of Ministry of Education on Food Synthetic Biotechnology, Jiangnan University, 1800 Lihu Road, Wuxi 214122, China

[5] Key Laboratory of Carbohydrate Chemistry and Biotechnology, Ministry of Education, Jiangnan University, 1800 Lihu Road, Wuxi 214122, China

[*] Correspondence: zhaoxinrui@jiangnan.edu.cn; jchen@jiangnan.edu.cn

Mailing address: Science Center for Future Foods, Jiangnan University, 1800 Lihu Road, Wuxi 214122, China

Supplementary Tables

Table S1. The strains applied and constructed in this study.

Strains	Description
DH5α	*E. coli* str. K-12 F⁻ *endA1 glnV44 thi-1 recA1 relA1 gyrA96 deoR nupG purB20* φ80d*lacZ*ΔM15 Δ(*lacZYA-argF*) U169 *hsdR17*($r_K^-m_K^+$) λ^-
BL21(DE3)	*E. coli* str. B F⁻ *ompT gal dcm lon hsdS*$_B$($r_B^-m_B^-$) λ (DE3 [*lacI lacUV5-T7p07 ind1 sam7 nin5*]) [*malB*$^+$]$_{K-12}$(λ^S)
S1 strain	*E. coli* BL21(DE3) harboring pRSFDuet-T7*lac-ho*^{T} plasmid
S2 strain	*E. coli* BL21(DE3) harboring pRSFDuet-T7*lac-ho*^{S} plasmid
S3 strain	*E. coli* BL21(DE3) harboring pRSFDuet-T7*lac-ho*^{N} plasmid
S4 strain	*E. coli* BL21(DE3) harboring pRSFDuet-T7*lac-ho*^{H} plasmid
S5 strain	*E. coli* BL21(DE3) harboring pRSFDuet-T7*lac-ho*^{NM} plasmid
S6 strain	*E. coli* BL21(DE3) harboring pRSFDuet-T7*lac-ho*^{B} plasmid
S7 strain	*E. coli* BL21(DE3) harboring pRSFDuet-T7*lac-ho*^{R} plasmid
S8 strain	*E. coli* BL21(DE3) harboring pRSFDuet-T7*lac-ho*^{O} plasmid
S9 strain	*E. coli* BL21(DE3) harboring pRSFDuet-T7*lac-ho*^{GM} plasmid
S10 strain	*E. coli* BL21(DE3) harboring pRSFDuet-T7*lac-ho*^{GG} plasmid
S11 strain	*E. coli* BL21(DE3) harboring pRSFDuet-T7*lac-ho*^{T}-T7*lac-pcyA*^{S} plasmid
S12 strain	*E. coli* BL21(DE3) harboring pRSFDuet-T7*lac-ho*^{T}-T7*lac-pcyA*^{SU} plasmid
S13 strain	*E. coli* BL21(DE3) harboring pRSFDuet-T7*lac-ho*^{T}-T7*lac-pcyA*^{P} plasmid
S14 strain	*E. coli* BL21(DE3) harboring pRSFDuet-T7*lac-ho*^{T}-T7*lac-pcyA*^{T} plasmid
S15 strain	*E. coli* BL21(DE3) harboring pRSFDuet-T7*lac-ho*^{T}-T7*lac-pcyA*^{N} plasmid

S16 strain	*E. coli* BL21(DE3) harboring pET28a (+)-T7-*iRFP* plasmid
S17 strain	*E. coli* BL21(DE3) harboring pET-28a (+)-T7-*Alr1966g2C56A* plasmid
S18 strain	*E. coli* BL21(DE3) harboring pRSFDuet-T7*lac-ADB1-ho1*^{T}(F29W/K166D)-T7*lac-ADB2-pcyA*^{S} *(D220G/H74M)* plasmid
S19 strain	*E. coli* BL21(DE3) harboring pRSFDuet-T7*lac-ADB1-ho1*^{T}*(F29W/K166D)*-T7*lac-ADB2-pcyA*^{S}*(D220G/H74M)* plasmid and pCDFDuet-T7*lac*-scaffold1-scaffold2-scaffold1 plasmid
S20 strain	*E. coli* BL21(DE3) harboring pRSFDuet-T7*lac-ADB1-ho1*^{T}*(F29W/K166D)*-T7*lac-ADB2-pcyA*^{S}*(D220G/H74M)* plasmid and pCDFDuet-T7*lac*-scaffold1-scaffold2-scaffold1-PnudC-*hemB*-*hemH* plasmid

Table S2. Genes used in this study.

Genes	Sequences
ho^S from *Synechocystis* sp. PCC6803	atgagcgttaacctggcgagccagctgcgtgaaggcaccaaaaaatctcactctatggcgga aaacgttggtttcgttaaatgcttcctgaaaggtgttgttgagaaaaacagctaccgtaaactgg ttggtaacctgtacttcgtttacagcgcgatggaagaagaaatggcgaaattcaaagatcaccc gatcctgtctcacatctacttcccggaactgaaccgtaaacagtctctggaacaggatctgcag ttctactatggtagcaactggcgtcaggaagttaaaatctctgcggcgggtcaggcgtacgttg atcgcgttcgtcaggttgcggcgaccgcgccggaactgctggttgcgcacagctacacccgt tacctgggcgcactgagcggcggccagatcctgaagaaaatcgcgcagaacgcgatgaac ctgcacgatggtggcaccgcgttctacgaattcgcagatatcgatgatgaaaaagcgttcaaa aacacctaccgtcaggctatgaacgatctgccgatcgatcaggcgaccgcggaacgtatcgt tgatgaagcgaacgatgcgttcgcgatgaacatgaaaatgttcaacgaactggaaggtaacct gatcaaagcgatcggcatcatggttttcaactctctgacccgtcgtcgtagccagggcagcac cgaagttggtctggcgacctctgaaggctaa
ho^N from *Nostoc sp.* PCC7120	atgagttcaaacttagctaataaactaagggtgggcaccaaaaaagcgcacacgatggcgga aaatgttggttttgtaaaatgctttctgaagggcgtcgtggaaaaaagcagctaccgcaagctg gttgctaacttttactacgtgtatagcgctatggaggaggaaatggagaagcattcgcaacatc cgattgtgagcaaaatcaacttcagccagttaaaccgtaagcagaccttggagcaggacctgt cttattactacggcgcaaattggagagaacaaatccagctgtctccggcaggcgaagcgtatg ttcagcgtattcgtgagatcagcgctaccgagccggaacttctgatcgcccactcctatacgcg ttatctgggcgacctgagcggtggtcaaattctgaagaacattgcagttaccgccatgaacctg aacgacggccaaggtacagccttctacgagttcgccgacatctccgatgagaaggcgttcaa

	agctaagtaccgccagaccctggatgagttggcgatcgacgaggcgacgggtgatcgtattg<br>ttgatgaggcgaacgcggcattcggcatgaatatgaaaatgtttcaagaattggaaggtaatct<br>catcaaagcgattggtatgatgctgtttaacacgctgacccgtaagcgcacccgtggtgcgac<br>tgagttggcgaccgcggaataa
ho^T from *Thermosynechococcus elongatus* BP-1	atgaccaccagcctggcgaccaaactgcgtgaaggcaccaaaaaagcgcacaccatggcg<br>gaaaacgttggcttcgttcgttgcttcctgaaaggcaccgttgaaaaagctcttaccgtaaact<br>ggttgcgagcctgtaccacgtttacagcgcgatggaacaggaaatggaacgtctgaaagatc<br>acccgatcgttggtaaaatctacttcccggaactgaaccgtaaatcttctctggaacgtgatctg<br>acctactacttcggctctaactggcgtgaagaaatcccgccgagcccggcgacccaggcgta<br>cgttgcgcgtatccacgaagttgcgaacaccgcgccggaactgctggttgcgcacagctaca<br>cccgttacctgggcgacctgagcggcggccagatcctgaaaggtatcgcggaacgtgcgat<br>gaacctgcaggatggcgaaggtaccgcgttctaccgtttcgaatccatcagcgatgaaaaag<br>cgttcaaacagctgtaccgtcagcgtctggatgaactgccggttgatgaagcgaccgcggat<br>cgtatcgttgatgaagcgaacgcggcgttcggtatgaacatgaaaatcttccaggaactggaa<br>ggtaacctgatccgtgcgatcggccagctgctgttcaacaccctgacccgtcgtaaacagcgt<br>ggcagcaccgaactggcgaccgcggattaa
ho^{NM} from *Neisseria meningitidis*	tctgaaaccgaaaaccaggcgctgaccttcgcgaaacgtctgaaagctgataccaccgcggt<br>tcacgatagcgttgataacctggttatgtctgttcagccgttcgtttctaaagaaaactatattaaa<br>ttcctgaaactgcagtctgttttccataaagctgttgatcatatctataaagatgcagaactgaac<br>aaagctatcccggaactggaatatatggcgcgttatgatgctgttacccaggatctgaaagatc<br>tgggtgaagaaccgtataaattcgataaaaaactgccgcacgaaaccggtaacaaagcgatt<br>ggttggctgtattgcgcggaaggtagcaacctgggtgcagcgttcctgttcaaacacgctcag

	aaactggattataacggtgaacacggcgctcgtcacctggctccgcacccggatggccgtgg
	taaacactggcgtgctttcgttgaacacctgaacgcgctgaacctgaccccggaagctgaag
	ctgaagcgatccagggtgcgcgtgaagcgttcgcattctacaaagttgttctgcgtgaaacctt
	cggtctggcggctgatgctgaagcgccggaaggtatgatgccgcaccgtcactaa
ho^{GM} from	atggcaagttcattatctacaatagctctattcctgccgcaaagcctgtacatcaaaccgagatt
Glycine max	ggcgccacgtccgctgccgccaccgcaatttcgctccctgtttttgacttctccggcgggtgca
	cgcaccgcgacggttatgccgcgtcgtgcagcggttattgtcagcgcggcgaccgcagaaa
	ctccgaaaaagaagggcgagtcgaaaggctttgtcgaggaaatgcgcttcgtggccatgcgt
	cttcacacccgtgatcaggcgcgtgaaggcgagaaagaagttaagcagccggaagaaaag
	gccgtgaccaaatgggacccgagcgtggaaggttacctgaaattcttggttgatagcaagttg
	gtttatgataccctggaaaagatcgtgcaagaggcaccgcacccgagctatgctgagttccgc
	aacaccggtttagagcgctctgcgagcctggcggaggacctggagtggttcaaagagcagg
	gttacaccattccggaaccgtctagcccgggtctgacgtacgcccagtatctgaaagaactgt
	ccgtgaaagaccctcaggcgtttatctgtcatttttacaacatttatttcgcgcatagcgctggcg
	gtcgtatgattggtaaaaaggtggctgagaagctgttgaataataaagcgctggagttctacaa
	gtgggatgacgacctgccgagattgctccaaaacgtgcgtgacaaactcaacaaggttgctg
	agccgtggtcacgtgaagagaaggaccactgcctggaggaaaccgaaaagagttttaagct
	gtccggtgagatcctgcgtcttatcctgtcctaa
ho^{H} from	atggaaaggccccaaccagattcaatgcctcaagacttgtccgaagcactgaaggaggcga
Homo	ccaaggaggttcacacccaggcggagaacgcggagtttatgcgtaatttccagaagggtcag
sapiens	gttacccgcgacggctttaagctggtgatggcgagcctgtaccatatttacgtggccctggag
	gaggagattgaacgtaataaagaaagcccggtgtttgcgccggtgtattttccggaggaacta

	caccgcaaagcagccttggaacaggatctggctttctggtatggtccgcgttggcaggaggt<br>gattccgtataccccagcaatgcaacgttacgtgaagcgtttgcacgaagtcggtcgtacgga<br>accggaattgctcgtagcgcacgcgtatactcgctacctgggtgatctaagcggtggccaggt<br>tctgaagaaaatcgcccagaaagcgctggacctgccgagcagcggtgaaggcctggcattc<br>ttcacctttccgaacatcgctagcgctaccaaattcaagcaactgtaccgctctcgtatgaatag<br>cctggaaatgaccccggcggtgcgacaacgtgtcatcgaggaggcgaaaacggcgttcctg<br>ctgaacatccagctgtttgaagagctgcaagagttgcttacccatgataccaaggaccagagc<br>ccgtctcgtgcgccaggtctgcgtcaacgcgcgtccaacaaagttcaggactccgcaccggt<br>tgaaacgccgcgtggcaaaccgcctctgaacaccagatcgcaagctccgctgttgcgctgg<br>gttctgaccctgtcattcctggtcgctactgtggctgttggcctgtacgcaatgtaa
ho^{B} from *Bos taurus*	atggaacgtccgcagccggatagctctatgccgcaggatctgtctgaagcgctgaaagaagc<br>gaccaaagaagttcacacccaggcggaaaacgcggaattcatgaaaaacttccagaaaggc<br>gaactgacccaggaaggtttcaaactggttatggctagcctgtaccacatctacgttgcgctgg<br>aagaagaaatcgaacgtaacaaagaaaacccggtttacaccccgctgtacttcccggaagaa<br>ctgcaccgtcgtgcgagcctggaacaggatatggcgttctggtacggtccgcgttggcagga<br>agctatcccgtacacccaggcgaccaaacgttacgttcagcgtctgcaggaagttggtcgtac<br>cgaaccggaactgctggttgcgcacgcgtacacccgttacctgggcgatctgagcggtggtc<br>aggttctgaaaaagatcgcgcagaaagcgctgaacctgccgagctctggcgaaggtctggc<br>gttcttcaccttcccgaacatcgcgtccgcgaccaaattcaaacagctgtaccgcagccgtatg<br>aacaccctggaaatgaccccggaagttcgtcagcgtgttctggacgaagcgaaaaccgcgtt<br>cctgctgaacatccagctgttcgaagaactgcagggcctgctgacccagaaagcgaaagatc<br>acgatccgctgcaggcgccggaactgcaccgtcgtgcgggctctaaagtgcaggatctggc

	gccgaccaaagcgagccgtggcaaaccgcagccgagcgttctgtctcaggctccgctgctg
	cgttgggttctgaccctgagcttcctggttgcgaccgttgcggttggcctgtacgcgatgtaa
ho^O from	atggaaaggacacacccagattcactaccccaagacctgagcgaagcgcttaaggaagcta
Oryctolagus	cgaaggaggttcatacccaggcggaaaacgccgagttcatgaaaaactttcagaaaggcca
cuniculus	agtgaccgcagagggctttaaactggtgatggcgtccctgtaccacatctacgtggcgttgga
	ggaggagatcgagcacaataaagaaaacccggtgtatgcaccgctgtacttcccggaagaa
	ctgcatcgtaaaccggcgttggagctggatatggctttctggtatggtccgcgctggcaagaa
	accattccgtataccccggctactcgccgttacgttcagcgcctgcaacaggtgggtcgtgcg
	gagccggaactcctggtcgcgcacgcatatacgcgttacctgggcgatttgtctggtggccag
	gtcctgaagaagatcgcgcagaaggctctggatttaccgtcgagcggcgaaggtgttgccttc
	ttcacctttccgcatattgcctctgcgaccaagttcaagcagttatatcgcagccgtatgaatagc
	ctggagatgacccctagtgttcgtcaacgtgttgttgaagaggcgaaagcagcgttcttgttga
	acattcagctgtttgaagaactgcaagagttgttggtgcaagacgcgcaaggtcagtccccat
	cccaagctccgggtctgcgccagagaaccggtagccgcacccaggacagcactccggtcg
	agccgccacgtggtaaaagccaactgtcgtcccttagccaaccgccgctgctgcgttgggttt
	tgaccctgtcttttctggtggccacggttgcgatgggcctgtacgcaatgtaa
ho^R from	atggaaaggccccaactagatagtatgtcacaagacctgagcgaagcgctgaaagaggcga
Rattus	ccaaagaagttcacatccgtgcggagaacagcgagttcatgcgtaactttcagaagggtcaa
norvegicus	gtcagccgtgaaggttttaagctggttatggcgagcctgtaccacatctacactgcgctggaag
	aggagatcgaacgtaataaacaaaacccggtttatgctccgctgtacttcccggaggagttgc
	accgcagagccgcgttggaacaggacatggcgttctggtatggtccgcattggcaggaggc
	gattccgtataccccctgcgacgcagcattacgtgaagcgcctgcacgaagtaggtggcaccc

	atccggagctgttggtcgcacatgcatatacgcgttatctgggtgatctgtctggtggccaagt
	gctgaagaaaatcgcccagaaggcaatggccttaccgtccagcggcgaaggcctggccttc
	ttcacctttccgtccattgataacccgaccaagttcaagcagctgtaccgcgcgcgtatgaata
	ccctggaaatgacccccgaggtgaagcaccgtgttaccgaggaggcgaaaaccgcgtttttg
	ttgaatattgaactttttgaggagctgcaagcactcctaacggaagagcacaaagaccagtcc
	ccgtcgcaaactgagttcctgcgccagcgtccggctagcctcgtgcaggataccacctcagc
	cgaaaccccacgtggtaaaagccaaattagcaccagctcctctcaaaccccgctgctgcgct
	gggttctgaccttgagttttctgctcgctacggtggcagttggcatctacgctatgtaa
ho^G from	atggagacatcacaaccccacaatgctgaatccatgagccaagatttgtccgaattactgaaa
Gallus gallus	gaggctacgaaagaggtacatgagcaggctgagaacaccccgtttatgaaaaactttcagaa
	gggccaagtctctctgcacgagttcaagttggtcaccgctagcctgtacttcatctacagcgcg
	ctggaggaagaaattgaacgtaataaggacaacccggtgtacgcgccagtgtacttccctatg
	gaactgcaccgtaaagcggctctggagaaagacttggagtacttctatggtagcaattggcgt
	gctgagatcccgtgcccggaagcgacgcagaagtatgttgaacgcctgcacgtggtgggca
	aaaagcacccggaactcctcgtcgcacatgcgtatacccgttatctgggcgacctgagcggc
	ggtcaagttcttaagaaaattgcacagaaggcgttgcaactgccgagcaccggtgaaggtct
	ggccttcttcacctttgatggtgtgtctaacgccaccaaattcaagcagctgtaccgcagtcgta
	tgaacgcgttggagatggaccacgcaaccaaaaagcgcgtgctggaggaggcgaaaaag
	gccttcctgttaaatattcaggtttttgaagcgttgcaaaaactggtgtccaaatcccaagaaaac
	ggccatgcggttcagccgaaagcggaactgcgcacccgttctgttaataagagccatgagaa
	cagcccggcagcgggtaaggagagcgaaagaacttcacgtatgcaggcggatatgctgac
	caccagcccgctggttcgttggctgttggctttaggttttatcgccacgactgtagccgttggctt

	gtttgcaatgtaa
pcyA^S from *Synechocystis* sp. PCC6803	atggcggttaccgatctgtctctgaccaacagcagcctgatgccgaccctgaacccgatgatc<br>cagcagctggcgctggcgatcgcggcgtcttggcagagcctgccgctgaaaccgtaccagc<br>tgccggaagatctgggttacgttgaaggccgtctggaaggtgaaaaactggttatcgaaaacc<br>gttgctaccagaccccgcagttccgtaaaatgcacctggaactggcaaaagtgggcaaaggt<br>ctggatatcctgcactgcgttatgttcccggaaccgctgtacggcctgccgctgttcggttgcg<br>atatcgttgcgggtccgggtggtgttagcgcggctatcgcggatctgtctccgacccagtctga<br>tcgtcagctgccggcggcgtaccagaaatctctggcggaactgggtcagccggaattcgaac<br>agcagcgtgaactgccgccgtggggtgaaatcttctctgaatactgcctgttcattcgtccgtct<br>aacgttaccgaagaagaacgtttcgttcagcgtgttgttgatttcctgcagatccactgccacca<br>gagcatcgtggcggaaccgctgagcgaagcgcagaccctggaacaccgtcagggccagat<br>ccactactgccagcagcagcagaaaaacgataaaacccgtcgtgttctggaaaaagcgttcg<br>gcgaagcgtgggcggaacgttacatgtctcaggttctgttcgatgttatccagtaa
pcyA^T from *Thermosynechococcus elongatus* BP-1	atgggttcattaaggcaacatcagcacccccctaatacaacgtctggctgatcgcattgaggcg<br>atctggcaggcgtttttcccgctggctccgtatgcgctcccagaggacctgggctatgttgaag<br>gtaaattggagggcgagcgtttgaccattgaaaatcattgttatcaggcgcctccgttcagaaa<br>gctgcacctggagctggcgcgtgtaggcgaaagcctggacatcctgcactgcgtgatgttcc<br>cggaaccgcgttacgacctgccgatgttcggctgcgacttggtgggcggtcgtggtcagatta<br>gcgcagccatcgtggatttgtccccggtcaccggtcagctgccggcagcgtatacctgtgcg<br>ctgaacgctctcccgaaactgacgtttcgccaaccgcgtgaactgccaccgtggggtcatattt<br>ttagcccgttctgcatcttcatccgtccgcagggtgaggctgaggaacagcaatttctggaccg<br>catcggcgagtacctgactctgcactgccagctgtctcagcaggccgttccgacggaccatc

	cgcaagcggttattgccggtcaacgccagtactgccagcaacaacaacaaaacgataaaac<br>ccgtcgtgtcttggaaaaggcattcggggtgccgtgggcggagcgctacatgaccaccgttc<br>tatttgatgttccaccggtgtaa
pcyA^{N} from *Nostoc* sp. PCC7120	atgggttcattaacatctatacccagtctaagggaacaacagcacccgctgattcgtcagttgg<br>ctgactgtatcgaagaggtgtggcatcagcacctggacctgagcccgtatcacttaccagctg<br>aactgggttacgtagaaggtcgcctggagggcgaaaaactgacgatcgagaaccgttgctac<br>cagactccgcagtttcgtaaaatgcatctggagttggcgaaagttggtaacatgctggacatcc<br>tgcattgtgtgatgtttccgcgtccggagtatgatcttccgatgttcggctgcgatttggtcggtg<br>gccgtggccagatttctgcggcgattgccgacttgtccccggttcatctggatcgcaccctgcc<br>ggagagctataacagcgcgctgaccagcctgaacaccttgaatttttcccagccgcgtgaatt<br>gccggaatggggtaatatcttctcggacttctgcatcttcgtgagaccatctagcccggaggag<br>gaggccatgttcttgggccgcgtgcgcgagttcctccaggtccactgccaaggtgcaattgct<br>gccagcccggtgagcgcggaacagaaacaacaaatcctggcgggtcaacacaattactgct<br>ccaagcagcaacaaaacgacaagacccgtcgtgttctggagaaggcgtttggcgttgattgg<br>gcagaaaactacatgaccacggttctgtttgatctgccggaataa
pcyA^{SU} from *Synechococcus* sp.	atgcaaccaaaacccctagctccgcctcccggacaacacccgctggtgcaggcactcgcgg<br>cgagcatccgcagcgcgtgggcaggcctgccgggtctggagattttaccgtgcgacgagga<br>tctgcgttttattcaaggtcagctggatggtgaaggcttgtctattggtaacgagctgttccgttg<br>cattggcctccgtaagctgcatctggaagtggcgcgcctgggcaacggtcttcaaattctgca<br>ctccgtgtggttcccggacccgcactacgacttgccgatttttggtgctgacatcgtcgcgggt<br>ccggcgggcattagcgccgctatcgtggacctgtccccgacctctgatgctttgcccgagca<br>gctgatccagcgtctggaggcgcgtccgtggccagcgttccgccaggtgagagaattgcca

	gcgtggggtagcgcgatcttcagcaacaaagtttgtttcatccgtccggacggcgcagacga<br>ggaagcggcatttcaggaactggttagccattatctgcaagttatggctacctccgttatcgaag<br>cgaccccggagccgagcaccgcattaacgactgtccgtcgctatgaaggccaattgaattact<br>gcctgcaacaaaagcgcaatgataaaacccgtcgtgttctggagaaggcctttgacagcgcc<br>tgggctgaccgttacatcgatatgctgctgttcgataacccgccggaactgtaa
pcyA^{P} from *Prochlorococcus marinus*	atgttaagtaaatcattgacaaaaactaagctaatcgacccgctgattctgaccctgctgcaaaa<br>catcaaggtgcagcgttcgaagttaaatgatctgaactgcattgaggtggacccgaagttaagt<br>aatatcatcagcaacgaagaaggtaaagagctgtatatcgagaacgagttctacaaagcgaa<br>aggctttcgcaaactgcacatcgaggtggccgaatttagcaagtctctgaagatcctgcattgt<br>gtgtttttcccggacccgaaatacgacatcccaattttcggcatggatttggttaaagtaaatgaa<br>ctggtgagcgcggcgatcgttgatctgtccccgagcagcaagaaccagaatctgaagtacga<br>ccacctgttgtcccatattgataaaagcgtttttaaatccaaacgtgaaatcccgatttggggtaa<br>tattttcagcaagaacgtgttcttcgctagccttaagaacgagtctgagaagaatgcattctgca<br>aaatcgtggacaactatctgtctgttcttatccagctctcgcaaagcacgagcccggacagcg<br>attatgaaattattgaggaacgtattaactaccagaagaactactgcgtccaacaaatgaaaaa<br>cgagaagacctccttggttttgctgaagtacttcgacaaggtttgggttgatgaatatattaaaaa<br>agtgttgtttgatttttaa
MBP	atgaaaatcgaagaaggtaaactggtaatctggattaacggcgataaaggctataacggtctc<br>gctgaagtcggtaagaaattcgagaaagataccggaattaaagtcaccgttgagcatccggat<br>aaactggaagagaaattcccacaggttgcggcaactggcgatggccctgacattatcttctgg<br>gcacacgaccgctttggtggctacgctcaatctggcctgttggctgaaatcaccccggacaaa<br>gcgttccaggacaagctgtatccgtttacctgggatgccgtacgttacaacggcaagctgattg

	cttacccgatcgctgttgaagcgttatcgctgatttataacaaagatctgctgccgaacccgcca<br>aaaacctgggaagagatcccggcgctggataaagaactgaaagcgaaaggtaagagcgcg<br>ctgatgttcaacctgcaagaaccgtacttcacctggccgctgattgctgctgacggggggttatg<br>cgttcaagtatgaaaacggcaagtacgacattaaagacgtgggcgtggataacgctggcgcg<br>aaagcgggtctgaccttcctggttgacctgattaaaaacaaacacatgaatgcagacaccgatt<br>actccatcgcagaagctgcctttaataaaggcgaaacagcgatgaccatcaacggcccgtgg<br>gcatggtccaacatcgacaccagcaaagtgaattatggtgtaacggtactgccgaccttcaag<br>ggtcaaccatccaaaccgttcgttggcgtgctgagcgcaggtattaacgccgccagtccgaa<br>caaagagctggcaaaagagttcctcgaaaactatctgctgactgatgaaggtctggaagcggt<br>taataaagacaaaccgctgggtgccgtagcgctgaagtcttacgaggaagagttggtgaaag<br>atccgcgtattgccgccactatggaaaacgcccagaaaggtgaaatcatgccgaacatcccg<br>cagatgtccgctttctggtatgccgtgcgtactgcggtgatcaacgccgccagcggtcgtcag<br>actgtcgatgaagccctgaaagacgcgcagact
iRFP	gctgaaggtagtgttgctaggcagcctgacttattaacttgtgacgatgaacctattcatattcct<br>ggtgctattcaacctcatggtttattattagctttagctgctgatatgactattgttgctggtagtgat<br>aatttacctgaattaactggtttagctattggtgctttaattggtagaagtgctgctgatgtttttgat<br>agtgaaactcataatagattaactattgctttagctgaacctggtgctgctgttggtgctcctatta<br>ctgttggttttactatgagaaaagatgctggttttattggtagttggcatagacatgatcaattaatt<br>tttttagaattagaacctcctcaaagagatgttgctgaacctcaagcatttttttagaagaactaata<br>gtgctattagaagattacaagctgctgaaactttagaaagtgcttgtgctgctgctgctcaagaa<br>gttagaaaaattactggttttgatagagttatgatttatagatttgctagtgattttagtggtgaagtt<br>attgctgaagatagatgtgctgaagttgaaagtaaattaggtttacattatcctgctagtactgttc

	ctgctcaagctagaagattatatactattaatcctgttagaattattcctgatattaattatagacctgttcctgttactcctgatttaaatcctgttactggtagacctattgatttaagttttgctattttaagaagtgttagtcctgttcatttagaatttatgagaaatattggtatgcatggtactatgagtattagtatttaagaggtgaaagattatggggtttaattgtttgtcatcatagaactccttattatgttgatttagatggtagacaagcatgtgaattagttgctcaagttttagcttggcaaattggtgttatggaagaataa
Alr1966g2C56A	cgatcgcaattattcgcggaaatcaccgtcaaaattcgggaaacccttgatattgaagagattctgcatacatcagttcaagaagtgcagaaacttttgcaagcagaccgagtattgattttccgactctgggccgatggttcgggaacagtagtacaagaatcagtgttacctggctggcctgtggtactgggacaaaatatccttgaccctgcgtttcaaaaagattacgttgataaatatcgccaagggcgagtcagtgccatagtagatgttactaaagcagatattcaagaatgtcatcgagaatttttgcaaggatttggcgtaaaagctaacctcgtagtccctattctcaatcgggatggcatttggggtttgctgattgcccatcagtgtacaaatcctcgccattggaataactttgagttggaattattgcaacaactagccaaccaaatgggaattgctttatcccaaggacaattattggaactacaaactaggcaaagt
ADB1	atgcgtagcaatcgtcgtgatcataccgtgagcacccgtcagagcaatatt
ADB2	atggtgagcagccgtcgtagccatcgtcgtagcaatcgctgcagcaatcgt
scalfold 1	caagctagggag
scalfold 2	gacgagggggtg
nudC from *Escherichia coli* BL21(DE3)	ttttcgtttagcctcatgtacgattctttgccaggcttaatgccaaccagattgtagtaagccacgagcagatgcttacgtacatgtagccagcgatcaaccagtttgttacttcctctgacgcgctccgtcaggttatcgagctggttaagcatgattgactccgcaagtttgtattcaaaaactgctcagtgagaaatgtaaaaaccatgttaaacatgccagtgatgcaaaggtagtgcaagagct
hemB from	acagacttaatccaacgccctcgtcgcctgcgcaaatctcctgcgctgcgcgctatgtttgaag

Escherichia coli BL21(DE3)	agacaacacttagccttaacgacctggtgttgccgatctttgttgaagaagaaattgacgactac<br>aaagccgttgaagccatgccaggtgtgatgcgcattccagagaaacatctggcacgcgaaat<br>tgaacgcatcgccaacgccggtattcgttccgtgatgactttcggcatctctcaccataccgatg<br>aaaccggcagcgatgcctggcgggaagatggactggtggcgcgaatgtcgcgcatctgca<br>agcagaccgtgccagaaatgatcgtcatgtcagacacctgcttctgcgaatacacatctcacg<br>gtcactgcggtgtgctgtgcgagcatggcgtcgacaacgacgcgactctggaaaatttaggc<br>aagcaagccgtggttgcagctgctgcaggcgcagacttcatcgccccttctgccgcgatgga<br>cggccaggtacaggcgattcgccaggcgctggacgctgcgggctttaaagatacggcgatt<br>atgtcgtattcgaccaagttcgcctcttccttttatggtccgttccgtgaagctgccggaagcgc<br>attaaaaggcgaccgcaaaagctatcagatgaacccaatgaaccgtcgtgaggcgattcgtg<br>agtcactgctggatgaagcccagggcgcagactgtctgatggttaaacctgccggagcgtac<br>ctcgacatcgtgcgtgagctgcgtgaacgtactgaattgccgattggcgcgtatcaggtgagc<br>ggtgagtacgcgatgattaagttcgccgcgctggcgggtgctatagatgaagagaaagtcgt<br>gctcgaaagcttaggttcaattaagcgtgcgggtgcggatctgattttcagctactttgcgatgg<br>atttggctgagaagaagattctgcgttaa
hemH from *Escherichia coli* BL21(DE3)	cgtcagactaaaaccggtatcctgctggcaaacctgggtacgcccgatgcccccacacctga<br>agcggtaaaacgctatctgaaacaatttttaagcgacagacgcgtggttgatacctcacggttg<br>ttatggtggccattgctgcgcggcgtgattttgccgctgcgctcgccgcgtgtggcgaagctgt<br>atgcctctgtctggatggaaggtggctcgccgctgatggtttacagccgtcagcaacagcagg<br>cgctggcacaacgtttaccggagacgcccgtagcgctgggaatgagctacggctcgccatc<br>actggaaagcgccgtagatgaactcctggcagagcatgtagatcatattgtggtgctgccgctt<br>tatccgcaatactcctgttcaacggtcggtgcggtatgggatgaactggcacgcattctggcgc

	gcaaacgtagcattccggggatatcgtttattcgtgattacgctgataaccacgattacattaatg<br>cactggcgaacagcgtacgcgcttcttttgccaaacatggcgaaccggatctgctgctgctctc<br>ttatcatggcattccccagcgttatgcagatgaaggcgatgattacccgcaacgttgccgcaca<br>acgactcgcgaactggcttccgcactggggatggcaccggaaaaagtgatgatgacctttca<br>gtcgcgctttggtcgggaaccctggctgatgccttataccgacgaaacgctgaaaatgctcgg<br>agaaaaaggcgtaggtcatatacaggtgatgtgcccgggctttgctgcggattgtctggagac<br>gctggaagagattgccgagcaaaaccgtgaggtcttcctcggtgccggcgggaaaaaatat<br>gaatatattccagcgcttaatgccacgccggaacatattgaaatgatggctaatcttgttgccgc<br>gtatcgctaa

Table S3. The primers used in this study.

Primer	Sequence (5'-3')[a]
F1	TCTTAGTATATTAGTTAAGTATAAGAAGGAGATATACATATG AAAATCGAAGAAGGTAAACTGGTAATCTGGATTAACGGC
R1	CAGAACCAGAACCAGAACCAGAACCAGTCTGCGCGTCTT TCAGGGC
F2	GAAATAATTTTGTTTAACTTTAATAAGGAGATATACCATGGG TAAAATCGAAGAAGGTAAACTGGTAATCTGGATTAACGGC G
R2	CGGAGCTAGGGGTTTTGGTTGCCTACCTTCGATACCAGAA CCAGAACCAGAAC
F3	AAGGAGATATACCATGGGTAAAATCGAAGAAGGTAAACTG GTAATCTGGATTAACGGCGA
R3	TGATTTACTTAACCTACCTTCGATACCAGAACCAGAACCAG AACCAGAACCAGTCTGCGCGTCTTTCAGGGCT
F4	GTTCTGGTTCTGGTTCTGGTATCGAAGGTAGGAGTTCAAAC TTAGCTAATAAACTAAGGGTGGGCAC
R4	CCAGTTTACCTTCTTCGATTTTCATATGTATATCTCCTTCTTA TACTTAACTAATATACTAAGATGGGGAATTG
F5	GTTCTGGTTCTGGTTCTGGTATCGAAGGTAGGGAGACATCA CAACCCCACAATGCTGAATCCATG
F6	CAACCAAAACCCCTAGCTCCGCCTCCCGGACAACACCCGC

R5	ACCCATGGTATATCTCCTTATTAAAGTTAAACAAAATTATTTCTACAGGGGAATTG
F7	TCTGGTTCTGGTATCGAAGGTAGGTTAAGTAAATCATTGACAAAAACTAAGCTAATCGAC
R6	AGTTTACCTTCTTCGATTTTACCCATGGTATATCTCCTTATTAAAGTTAAACAAAATTATTTCTACAG
F8	ACCGTGAGCACCCGTCAGAGCAATATTGGCGGTGGCGGTAGCACGACTTCATTAGCTACAAAACT
R7	CTGACGGGTGCTCACGGTATGATCACGACGATTGCTACGCATATGTATATCTCCTTCTTATACTTAACT
F9	CGTCGTAGCAATCGCTGCAGCAATCGTGGCGGTGGCGGTAGCGCGGTTACCGATCTGTCT
R8	GCAGCGATTGCTACGACGATGGCTACGACGGCTGCTCACCATGGTATATCTCCTTATTAAAGTT
F10	CAAGCTAGGGAGAATCATAAGACGAGGGGGTGTATTCACGCAAGCTAGGGAGGGATCCGAATTCGAGCTC
R9	TTATGATTCTCCCTAGCTTGATTAAAGTTAAACAAAATTATTTCTACAG
F11	TGCAAGAGCTCATATGGCAGATCTCAATTGG
R10	TAAACGAAAAGATTATGCGGCCGTGTAC
F12	CCGCATAATCTTTTCGTTTAGCCTCATGTACGAT
R11	CTGCCATATGAGCTCTTGCACTACCTTTGCATC

F13	AAGAGCTCATATGACAGACTTAATCCAACGCC
R12	TGAGATCTTTAACGCAGAATCTTCTTCTCAG
F14	AAGAAGATTCTGCGTTAAAGATCTATGCGTCAGACTAAAA CCGGT
R13	AGACTCGAGTTAGCGATACGCGGCAAC

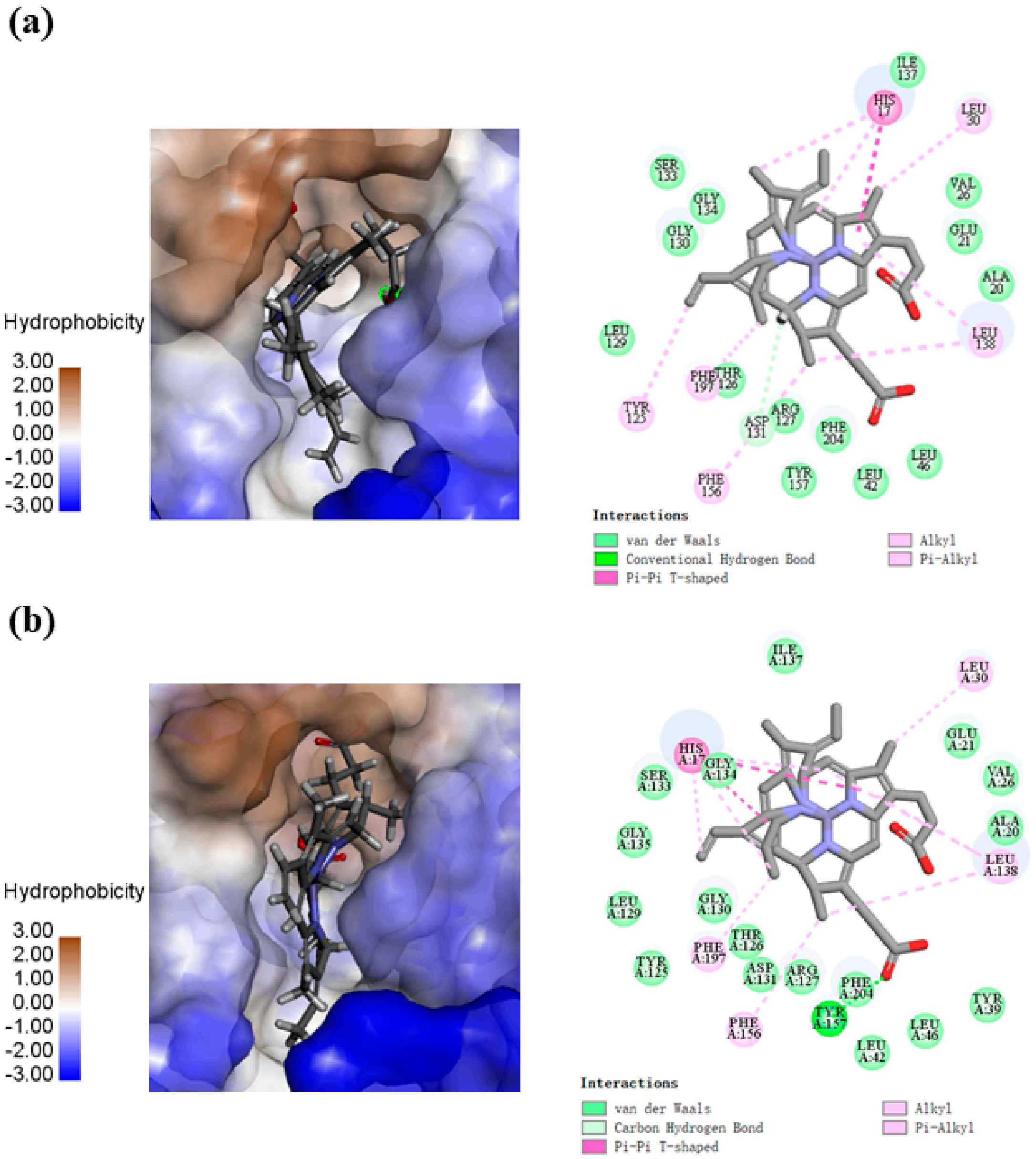

Figure S1. The interactions between heme and HO^T model (**a**) and HO^T (F29W/K166D) model (**b**).

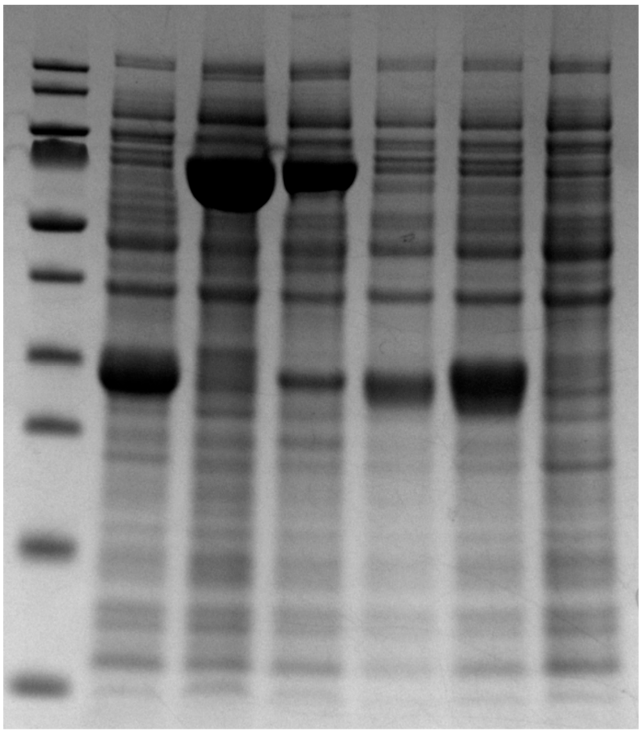

Figure S2. SDS-PAGE results of S11-S15 strains (the original image of Figure 5c).

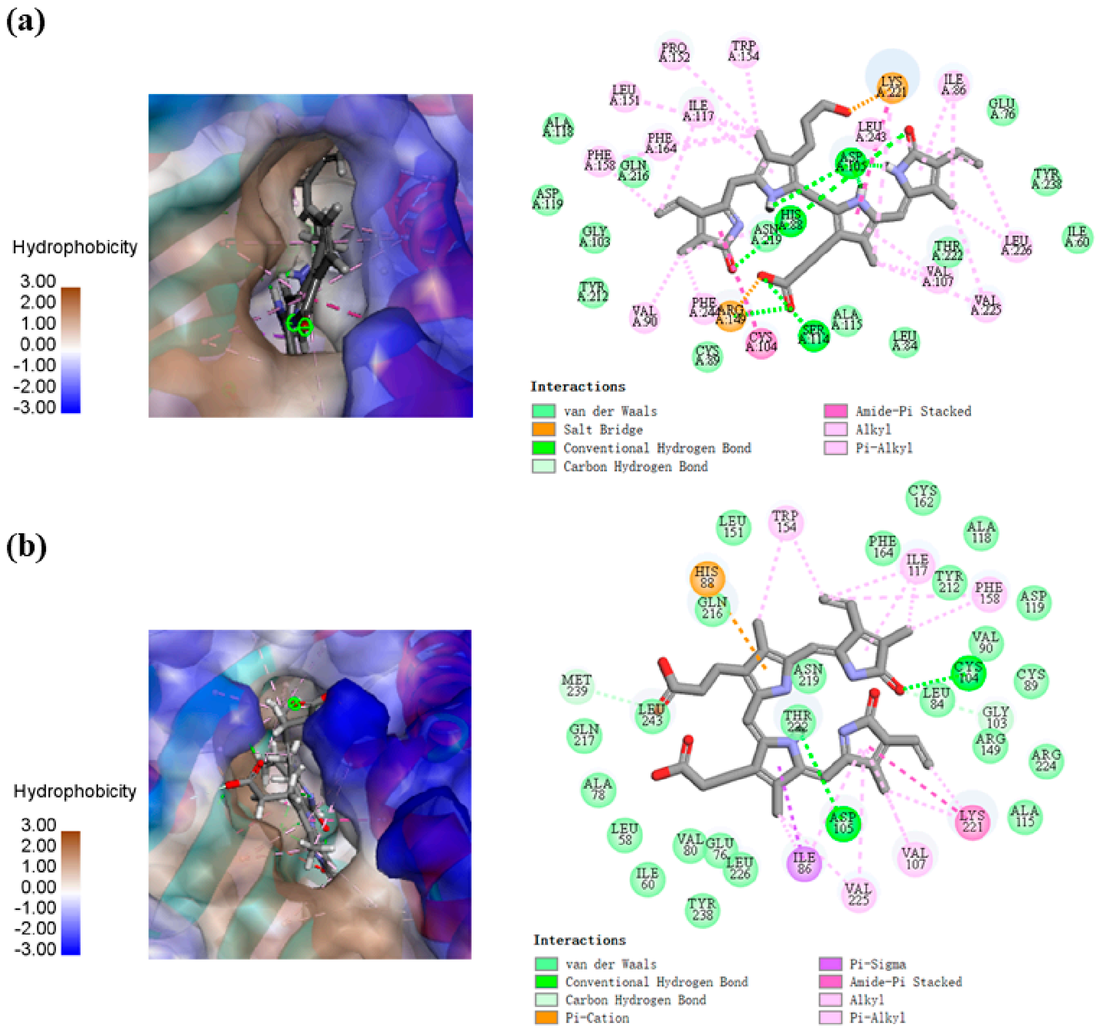

Figure S3. The interactions between BV and PcyA^{S} model (**a**) and PcyA^{S} (D220G/H74M) model (**b**).
